# Incidence and risk of venous thromboembolism according to primary treatment type in women with endometrial cancer: a population-based study

**DOI:** 10.1186/s12885-021-08853-x

**Published:** 2021-10-30

**Authors:** Jin-Sung Yuk, Banghyun Lee, Kidong Kim, Myoung Hwan Kim, Yong-Soo Seo, Sung Ook Hwang, Yong Kyoon Cho, Yong Beom Kim

**Affiliations:** 1 Department of Obstetrics and Gynecology, Sanggye Paik Hospital, School of Medicine, Inje University, Seoul, Republic of Korea; 2 Department of Obstetrics and Gynecology, Inha University hospital, Inha University School of Medicine, 27, Inhang-ro, Sinheung-dong, Jung-gu, Incheon, Republic of Korea; 3 Department of Obstetrics and Gynecology, Seoul National University Bundang Hospital, Seongnam-Si, Gyeonggi-Do Republic of Korea

**Keywords:** Chemotherapy, Endometrial cancer, Hormone therapy, Surgery, Radiotherapy, Venous thromboembolism

## Abstract

**Background:**

Current prophylaxes and treatments for venous thromboembolism (VTE) in women with gynecologic cancer are mainly guided by studies on solid cancers because studies in gynecologic cancer did not provide sufficient data. Large-scale studies evaluating the incidence and risk of VTE according to therapeutic modality may guide prophylaxis and treatment of VTE in gynecologic cancer. This study was performed to determine the incidence and risk of VTE according to primary treatment type in Korean women with endometrial cancer.

**Methods:**

We selected 26,256 women newly diagnosed with endometrial cancer between 2009 and 2018 from the Korean Health Insurance Review and Assessment Service database. During the total follow-up period and first six months after primary treatments initiation, the incidence and risk of VTE were evaluated according to primary treatment type, that is, no treatment, surgery, radiotherapy, chemotherapy, or hormone therapy.

**Results:**

VTE occurred in 136 per 10,000 women during the total follow-up period and in 54 per 10,000 women during the first six months with the highest frequency in women that underwent chemotherapy. During the first year, the monthly incidence of VTE decreased with time among women that underwent no treatment, surgery, or hormone therapy and remained unchanged in those that received radiotherapy or chemotherapy. Compared with women that received no treatment, VTE risk, especially of PE significantly increased in women that underwent chemotherapy (VTE: hazard ratio (HR), 2.334; 95% CI, 1.38–3.949; *P* = 0.002) (PE: HR, 2.742; 95% CI, 1.424–5.278; *P* = 0.003) or hormone therapy (VTE: HR, 2.073; 95% CI, 1.356–3.17; *P* = 0.001) (PE: HR, 2.086; 95% CI, 1.19–3.657; *P* = 0.01) during the total follow-up period and women that underwent only chemotherapy during the first six months (VTE: HR, 2.532; 95% CI, 1.291–4.966; *P* = 0.007) (PE: HR, 3.366; 95% CI, 1.496–7.576; *P* = 0.003).

**Conclusions:**

In this cohort study, the incidence and risk of VTE were highest in women with endometrial cancer that underwent chemotherapy as a primary treatment. Notably, the incidence of VTE decreased over time in women that received no treatment, surgery, or hormone therapy. This study can help guide therapies for prophylaxis and treatment of VTE in women with endometrial cancer.

**Supplementary Information:**

The online version contains supplementary material available at 10.1186/s12885-021-08853-x.

## Introduction

Endometrial cancer is the most common cancer of the female reproductive tract [1], and its incidence has increased due to increases in elderly and obese populations [2]. In addition to hypercoagulability, which is characteristic of cancer, factors such as old age, obesity, surgery, radiotherapy, chemotherapy, and hormone therapy can induce venous thromboembolism (VTE) in women with cancer, including gynecologic cancers [3, 4].

VTE, which includes deep vein thrombosis (DVT) and pulmonary embolism (PE), is a common complication of gynecologic cancers [3]. The incidence of VTE in women with gynecologic cancer depends on the type of treatment received (e.g., surgery, radiotherapy, or chemotherapy) [5–19].

When endometrial cancer is suitable for surgery and does not have distant metastasis, the primary treatment comprises total hysterectomy (TH) or radical hysterectomy (RH), bilateral salpingo-oophorectomy (BSO) and lymph node assessment [20]. When endometrial cancer is unsuitable for primary surgery or is accompanied by distant metastasis, primary treatments include external beam radiation therapy (EBRT), brachytherapy, chemotherapy, hormone therapy, or palliative TH and BSO [20]. However, although there are various treatments for endometrial cancer, relatively few studies have evaluated the relationship between VTE and therapeutic modality [6, 7, 9–13, 15, 16].

Currently, most prophylaxes and treatments for VTE in women with gynecologic cancer are guided by studies on solid cancers, such as lung, stomach, pancreatic, brain, and renal cancer, because studies in gynecologic cancer have not provided sufficient data [3, 4, 21, 22]. We considered that conducting a large-scale study on the incidence and risk of VTE according to therapeutic modality might guide prophylaxis and treatment of VTE in endometrial cancer. Therefore, we evaluated the incidence and risk for VTE according to primary treatment type in women with endometrial cancer using Korean national health insurance data.

## Materials and methods

Approximately 98% of the Korean population is covered by a universal health coverage system (National Health Insurance) [23]. The claims data of the Health Insurance Review and Assessment Service (HIRA) include 23 million women per year [23]. In the present study, we used claims data from the HIRA database of women with diagnostic codes for endometrial cancer generated between January 1, 2007, and December 31, 2018. Approval for this study was waived by the Institutional Review Board of Inha University Hospital (No. 2019–11-007) on November 25, 2019, because the HIRA dataset uses anonymous identification codes to protect personal information, as is required by the Bioethics and Safety Act in South Korea. Informed consent was not required.

The diagnostic, surgical, and prescription codes used to select eligible women were based on the 10th revision of the International Statistical Classification of Diseases and Related Health Problems (ICD-10), Health Insurance Medical Care Expenses (2017 and 2018 version), and HIRA Drug Ingredients Codes. Using the Korea Central Cancer Registry as a reference, women with endometrial cancer were defined as women with five or more diagnostic codes for endometrial cancer (ICD-10: C54x or C55x) and no diagnostic code for any other cancer between 2007 and 2018. Among women diagnosed with endometrial cancer, women with diagnostic codes for VTE (ICD-10: I80.2, I80.3, I26.0, I26.9) before the first C54x or C55x codes were excluded. Women with C54x or C55x codes between 2007 and 2008 (the wash out period) were also excluded to extract only women with newly diagnosed endometrial cancer. Women with VTE were defined as women that received prescriptions for anticoagulants more than twice simultaneously with diagnostic codes for VTE after the initiation of primary cancer treatment (surgery, radiotherapy, chemotherapy, or hormone therapy). Women who did not receive primary cancer treatment were defined as having VTE if they received prescriptions for anticoagulants more than twice simultaneously with diagnostic codes for VTE from the date of the first diagnostic code for endometrial cancer. DVT and PE cases were defined based on the receipt of more than two prescriptions for anticoagulants under diagnostic codes of I80.2 or I80.3 and I26.0 or I26.9, respectively.

Subject ages were categorized using intervals of 5 years. Low socioeconomic status (SES) was defined as the use of Medicaid as National Health Insurance. Charlson Comorbidity Indices (CCIs) were calculated using data obtained between 365 days and 1 day before the first diagnostic date of endometrial cancer, as described by Quan [24]. Primary treatments (no treatment, surgery, radiotherapy, chemotherapy, and hormone therapy) were defined as treatments performed first after diagnosis of endometrial cancer. In the no treatment group, the incidence of VTE after diagnosis of endometrial cancer was evaluated. No treatment was defined as the lack of a prescription code for surgery, radiotherapy, or chemotherapy; women in this category, were diagnosed with endometrial cancer but did not receive surgery, radiotherapy, or chemotherapy.

Surgery was defined using surgery codes for TH or RH and the presence of simultaneous diagnostic codes for endometrial cancer. If two or more surgeries were performed, the first surgery was defined as the primary treatment. Radiotherapy was defined as prescription codes for concurrent chemoradiotherapy (CCRT), EBRT (external beam radiation therapy) or brachytherapy with simultaneous diagnostic codes for endometrial cancer. CCRT was defined as codes for EBRT and simultaneous codes for chemotherapy. Chemotherapy was defined as prescription codes for chemotherapy (cisplatin, carboplatin, dacarbazine, docetaxel, doxorubicin, epirubicin, everolimus, gemcitabine, ifosfamide, liposomal doxorubicin, paclitaxel, pazopanib, trabectedin, temozolomide, temsirolimus, topotecan, or bevacizumab) with a simultaneous diagnostic code for endometrial cancer. Hormone therapy was defined as prescription codes for hormone therapy (progestins, tamoxifen, aromatase inhibitors, gonadotropin-releasing hormone agonists, levonorgestrel-releasing intrauterine system) with a simultaneous diagnostic code for endometrial cancer. Use of prophylactic anticoagulants for VTE was defined as the receipt of anticoagulants more than twice without diagnostic codes for VTE after the initiation of primary treatment or from the date of endometrial cancer diagnosis (in the no treatment group). Prescribed anticoagulants included aspirin, direct oral anticoagulants (DOAC), fondaparinux, low molecular weight heparin (LMWH), unfractionated heparin (UFH), and warfarin.

### Statistical analyses

The independent t-test and Mann-Whitney U test were used for continuous variables, whereas the Chi-square test and Fisher’s exact test were used for categorical variables. The Cochran-Armitage trend test was used for trend analysis, and Cox Proportional Hazard Regression analysis with or without adjustment for confounders was used to analyze associations between variables and VTE. Two-tailed tests were used throughout, and *P* values of < 0.05 were considered statistically significant. The mean imputation method was used to account for missing values. For analyses, SAS® Enterprise Guide® version 6.1 (SAS Institute, Inc., Cary, NC, USA) and R version 3.3.2 (R Foundation for Statistical Computing, Vienna, Austria) were used.

## Results

**Fig. 1:**
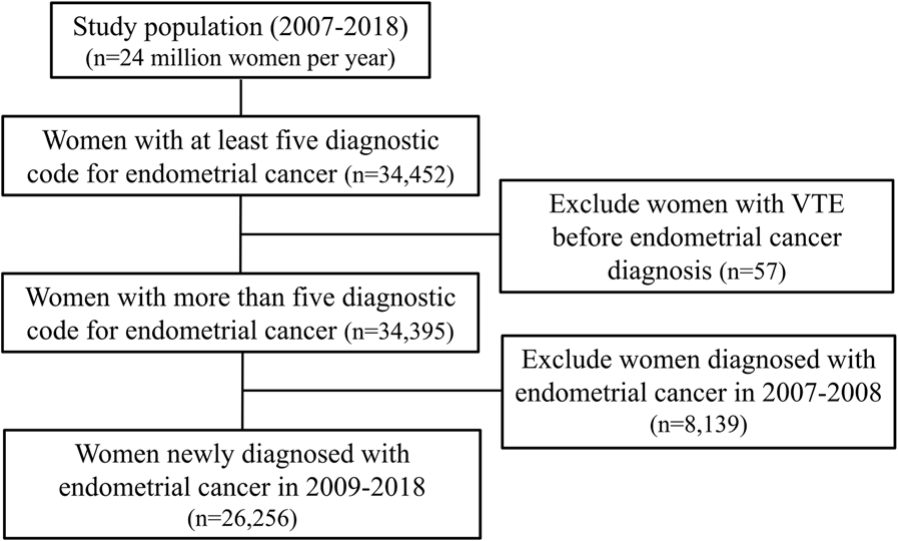
Flow chart of the study selection procedure

A schematic of the selection process is provided in Fig. 1. Initially, the data of 34,452 women with five or more diagnostic codes for endometrial cancer between 2007 and 2018 were extracted, and 26,256 women with newly diagnosed endometrial cancer from 2009 were finally selected.

### Baseline characteristics of women with endometrial cancer

The 26,256 women with endometrial cancer included were followed up for an average of 1418.9 ± 6.3 days. Detailed characteristics of the study subjects are provided in Table 1.

**Table 1:** Characteristics of women with endometrial cancer

	The total follow-up period				The first six months			
	VTE (−)	VTE (+)	Total		VTE (−)	VTE (+)	Total	
	*n* = 25,899 (98.6%)	*n* = 357 (1.4%)	*n* = 26,256 (100%)	*P* value	*n* = 26,114 (99.5%)	*n* = 142 (0.5%)	*n* = 26,256 (100%)	*P* value
Mean age (years), mean ± SE	54.17 ± 0.1	59.25 ± 0.6	54.24 ± 0.1	<.001 ^a^	54.21 ± 0.1	58.35 ± 0.9	54.24 ± 0.1	<.001 ^a^
SES, n (%)				0.101				0.61
Mid- or high- SES	24,767 (95.6)	335 (93.8)	25,102 (95.6)		24,965 (95.6)	137 (96.5)	25,102 (95.6)	
Low SES	1132 (4.4)	22 (6.2)	1154 (4.4)		1149 (4.4)	5 (3.5)	1154 (4.4)	
CCI, n (%)				<.001				0.695
0	14,299 (55.2)	159 (44.5)	14,458 (55.1)		14,387 (55.1)	71 (50)	14,458 (55.1)	
1	3464 (13.4)	48 (13.4)	3512 (13.4)		3491 (13.4)	21 (14.8)	3512 (13.4)	
2	4688 (18.1)	76 (21.3)	4764 (18.1)		4738 (18.1)	26 (18.3)	4764 (18.1)	
3	1366 (5.3)	24 (6.7)	1390 (5.3)		1381 (5.3)	9 (6.3)	1390 (5.3)	
Over 4	2082 (8)	50 (14)	2132 (8.1)		2117 (8.1)	15 (10.6)	2132 (8.1)	
Year of endometrial cancer diagnosis, n (%)				0.101				0.016
2009	1991 (7.7)	20 (5.6)	2011 (7.7)		2007 (7.7)	4 (2.8)	2011 (7.7)	
2010	2099 (8.1)	25 (7)	2124 (8.1)		2116 (8.1)	8 (5.6)	2124 (8.1)	
2011	2238 (8.6)	45 (12.6)	2283 (8.7)		2268 (8.7)	15 (10.6)	2283 (8.7)	
2012	2319 (9)	32 (9)	2351 (9)		2342 (9)	9 (6.3)	2351 (9)	
2013	2508 (9.7)	37 (10.4)	2545 (9.7)		2537 (9.7)	8 (5.6)	2545 (9.7)	
2014	2553 (9.9)	38 (10.6)	2591 (9.9)		2583 (9.9)	8 (5.6)	2591 (9.9)	
2015	2686 (10.4)	42 (11.8)	2728 (10.4)		2707 (10.4)	21 (14.8)	2728 (10.4)	
2016	3081 (11.9)	47 (13.2)	3128 (11.9)		3106 (11.9)	22 (30)	3128 (11.9)	
2017	3326 (12.8)	41 (11.5)	3367 (12.8)		3342 (12.8)	25 (17.6)	3367 (12.8)	
2018	3098 (12)	30 (8.4)	3128 (11.9)		3106 (11.9)	22 (15.5)	3128 (11.9)	
Primary treatments ^b^, n (%)				0.1				< 0.001
No treatement ^c^	5268 (20.3)	70 (19.6)	5338 (20.3)		5307 (20.3)	31 (20)	5338 (20.3)	
Surgery ^d^	17,386 (67.1)	231 (64.7)	17,617 (67.1)		17,509 (67.1)	108 (69.7)	17,617 (67.1)	
Radiotherapy ± chemotherapy	544 (2.1)	6 (1.7)	550 (2.1)		546 (2.1)	4 (2.6)	550 (2.1)	
Chemotherapy	723 (2.8)	18 (5)	741 (2.8)		729 (2.8)	12 (7.7)	741 (2.8)	
Hormone therapy	1978 (7.6)	32 (9)	2010 (7.7)		2010 (7.7)	0 (0)	2010 (7.7)	
Surgery, n (%)								
Total hysterectomy	9874 (56.8)	113 (48.9)	9987 (56.7)	0.016	9932 (56.7)	55 (50.9)	9987 (56.7)	0.225
Radical hysterectomy	7512 (43.2)	118 (51.1)	7630 (43.3)	0.016	7577 (43.3)	53 (49.1)	7630 (43.3)	0.225
Radiotherapy, n (%)								
CCRT	16 (2.9)	1 (16.7)	17 (0)	0.172 ^e^	17 (3.1)	0 (0)	17 (0)	1 ^e^
EBRT	306 (56.3)	5 (83.3)	311 (86.9)	0.24 ^e^	308 (56.4)	3 (75)	311 (86.9)	0.637 ^e^
Brachytherapy	313 (57.5)	2 (33.3)	315 (37.4)	0.41 ^e^	314 (57.5)	1 (25)	315 (37.4)	0.318 ^e^
Chemotherapy, n (%)								
Platinum (cisplatin, carboplatin)	203 (48.2)	11 (61.1)	214 (48.7)	0.442	506 (69.6)	8 (66.7)	514 (69.6)	0.764 ^e^
Other agents	218 (51.8)	7 (38.9)	225 (51.3)	0.426	221 (30.4)	4 (33.3)	225 (30.4)	0.761 ^e^
Bevacizumab	16 (3.8)	0 (0)	16 (3.6)	1 ^e^	16 (2.2)	0 (0)	16 (2.3)	1 ^e^
Hormone therapy								
Progestins	1823 (74.6)	29 (72.5)	1852 (74.5)	0.736 ^e^	1852 (74.5)	0 (0)	1852 (74.5)	1 ^e^
Tamoxifen	49 (2)	2 (5)	51 (2.1)	0.194 ^e^	51 (2.1)	0 (0)	51 (2.1)	1 ^e^
Progestins and Tamoxifen	33 (1.3)	2 (5)	35 (1.4)	0.106 ^e^	35 (1.4)	0 (0)	35 (1.4)	1 ^e^
Aromatase inhibitors	69 (2.8)	4 (10)	73 (2.9)	0.027 ^e^	73 (2.9)	0 (0)	73 (2.9)	1 ^e^
Gonadotropin-releasing hormone agonists	21 (0.9)	1 (2.5)	22 (0.9)	0.299 ^e^	22 (0.9)	0 (0)	22 (0.9)	1 ^e^
Levonorgestrel-releasing intrauterine system	450 (18.4)	2 (5)	452 (18.2)	0.027	452 (18.2)	0 (0)	452 (18.2)	1 ^e^
Time between primary treatments and VTE diagnosis (days), mean ± SE		526.8 ± 46.4				79.9 ± 7.5		

*CCI* Charlson comorbidity index; *CCRT* concurrent chemoradiation therapy; *EBRT* external beam radiation therapy; *HIRA* health insurance review & assessment Service; *n* number; *SES* socioeconomic status; *VTE* venous thromboembolism

All values are expressed as mean ± standard error or number (%)

^a^ The Mann-Whitney U test was used for this analysis

^b^ Primary treatments refers to the first cancer treatments

^c^ Incidence of VTE after diagnosis of endometrial cancer was evaluated

^d^ Neoadjuvant chemotherapy followed by laparotomy or laparoscopy was considered as surgery

^e^ The Fisher’s exact test was used for this analysis

### Incidences of VTE according to primary treatment modality types

During the total follow-up period after primary treatment initiation, VTE occurred in 136 per 10,000 women, with treatment modalities in order of decreasing frequency as chemotherapy, hormone therapy, no treatment and surgery, and radiotherapy. During the first 6 months after primary treatment initiation, VTE occurred in 54 per 10,000 women, with treatments in decreasing order of frequency as chemotherapy, radiotherapy, surgery, and no treatment. VTE was not observed in the hormone therapy group during the first 6 months (Table 2).

**Table 2:** Incidences (per 10,000 women) of VTE according to primary treatments

	The total follow-up period						The first six months						Total cases
	VTE		DVT		PE		VTE		DVT		PE		
	Count	Incidence	Count	Incidence	Count	Incidence	Count	Incidence	Count	Incidence	Count	Incidence	
Primary treatments ^a^													
No treatment ^b^	70	131	33	62	41	77	31	58	13	24	18	34	5338
Surgery ^c^	231	131	120	68	127	72	108	61	50	28	63	36	17,617
Radiotherapy	6	109	4	73	3	55	4	73	3	55	2	36	550
Chemotherapy	18	243	7	94	12	162	12	162	3	40	9	121	741
Hormone therapy	32	159	14	70	18	90	0	0	0	0	0	0	2010
Total cases	357	136	178	68	201	77	142	54	64	24	84	32	26,256

*DVT* deep vein thrombosis; *HIRA* Health Insurance Review & Assessment Service; *PE* pulmonary embolism; *VTE* venous thromboembolism

^a^ Primary treatments refers to the first cancer treatments

^b^ Incidence of VTE after diagnosis of endometrial cancer was evaluated

^c^ Neoadjuvant chemotherapy followed by laparotomy or laparoscopy was considered as surgery

**Fig. 2:**
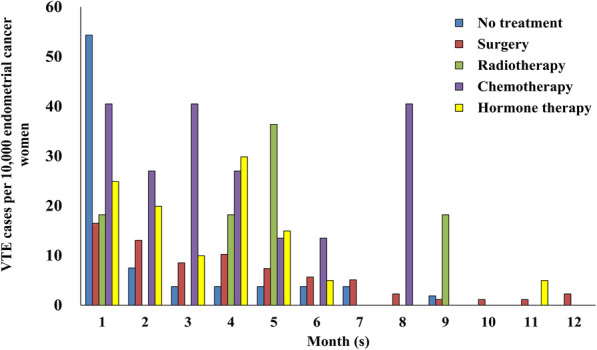
Monthly incidences of VTE during the first year after primary treatment initiation. In women that received no treatment, the incidence of VTE after diagnosis of endometrial cancer was evaluated

During the first year after primary treatment initiation, the monthly incidence of VTE decreased over time in the no treatment, surgery, and hormone therapy groups (Cochran-Armitage trend test: *P* < 0.001), but did not change with time in the radiotherapy or chemotherapy groups (Fig. 2).

During the total follow-up period, the incidence of VTE increased according to increase of age in the no treatment, surgery, and hormone therapy groups (Cochran-Armitage trend test: *P* < 0.001), but no age-related change was observed in the radiotherapy or chemotherapy group. The incidence of VTE in the surgery group was highest for women aged 20–25 years and in the chemotherapy group was highest for women aged 30–35 years. Furthermore, the incidences of VTE among women aged 25–30 years that underwent surgery and among those aged 35–40 years that underwent chemotherapy were higher than for women of other ages. These findings may have been due to the relatively small numbers of women with endometrial cancer in these age groups. The incidence of VTE began to decrease at 70 or 75 years of age for all types of primary treatment, except hormone therapy (Additional files 1 and 2).

### Relationship between risk factors and VTE occurrence

During the total follow-up period, the risk of VTE significantly increased according to increased age, CCI, and year of endometrial cancer diagnosis (age: hazard ratio (HR), 1.231; 95% CI, 1.175–1.29; *P* < 0.001) (CCI: HR, 1.121; 95% CI, 1.071–1.173; *P* < 0.001) (year of endometrial cancer diagnosis: HR, 1.112; 95% CI, 1.064–1.162; *P* < 0.001). A low SES was not a risk factor of VTE. Receipt of chemotherapy or hormone therapy as primary treatments were associated with significant increases in VTE risk, especially of PE, as compared with no treatment after adjusting for confounders (chemotherapy: VTE: HR, 2.334; 95% CI, 1.38–3.949; *P* = 0.002) (chemotherapy: PE: HR, 2.742; 95% CI, 1.424–5.278; *P* = 0.003) (hormone therapy: VTE: HR, 2.073; 95% CI, 1.356–3.17; *P* = .001) (hormone therapy: PE: HR, 2.086; 95% CI, 1.19–3.657; *P* = 0.01), but surgery and radiotherapy were not found to influence VTE risk as compared with no treatment (Table 3).

**Table 3:** Association of risk factors with VTE occurrence

	The total follow-up period						The first six months					
	VTE		DVE		PE		VTE		DVE		PE	
	HR (95% CI)	*P* value	HR (95% CI)	*P* value	HR (95% CI)	*P* value	HR (95% CI)	*P* value	HR (95% CI)	*P* value	HR (95% CI)	*P* value
Unadjusted HR												
Age per 5 years	1.244 (1.187–1.303)	< 0.001	1.205 (1.128–1.287)	< 0.001	1.294 (1.216–1.378)	< 0.001	1.175 (1.096–1.258)	< 0.001	1.151 (1.038–1.275)	0.008	1.177 (1.076–1.287)	< 0.001
Low SES ^a^	1.547 (1.005–2.381)	0.048	1.413 (0.747–2.675)	0.288	1.756 (1.02–3.023)	0.042	0.889 (0.393–2.011)	0.778	0.325 (0.045–2.338)	0.264	1.27 (0.515–3.127)	0.604
CCI	1.159 (1.112–1.208)	< 0.001	1.148 (1.081–1.22)	< 0.001	1.164 (1.102–1.229)	< 0.001	1.073 (0.999–1.153)	0.054	1.051 (0.938–1.176)	0.392	1.078 (0.983–1.182)	0.603
Year of endometrial cancer diagnosis	1.116 (1.069–1.165)	< 0.001	1.118 (1.051–1.19)	< 0.001	1.109 (1.048–1.174)	< 0.001	1.138 (1.071–1.209)	< 0.001	1.145 (1.045–1.254)	0.004	1.129 (1.045–1.221)	0.002
Types of primary treatment ^b,c^												
Surgery ^d^	0.91 (0.696–1.189)	0.489	0.995 (0.677–1.463)	0.979	0.857 (0.603–1.219)	0.391	1.03 (0.691–1.535)	0.885	1.137 (0.618–2.093)	0.68	1.034 (0.613–1.747)	0.899
Radiotherapy	0.79 (0.343–1.818)	0.579	1.11 (0.393–3.134)	0.843	0.676 (0.209–2.182)	0.512	1.26 (0.446–3.576)	0.661	2.259 (0.644–7.927)	0.203	1.087 (0.252–4.684)	0.911
Chemotherapy	2.614 (1.554–4.395)	< 0.001	2.234 (0.986–5.063)	0.054	2.934 (1.538–5.596)	0.001	2.956 (1.518–5.757)	0.001	1.756 (0.5–6.163)	0.379	3.827 (1.719–8.521)	0.001
Hormone therapy	1.261 (0.83–1.915)	0.278	1.173 (0.628–2.192)	0.617	1.207 (0.693–2.101)	0.506	0 (0-Infinite)	0.991	0 (0-Infinite)	0.994	0 (0-Infinite)	0.991
Adjusted HR ^e^												
Age per 5 years	1.231 (1.175–1.29)	< 0.001	1.191 (1.114–1.273)	< 0.001	1.279 (1.201–1.363)	< 0.001	1.147 (1.066–1.235)	< 0.001	1.125 (1.006–1.257)	0.038	1.148 (1.043–1.263)	0.005
Low SES ^a^	1.119 (0.723–1.734)	0.614	1.075 (0.564–2.052)	0.826	1.205 (0.694–2.094)	0.507	0.722 (0.317–1.646)	0.439	0.271 (0.037–1.966)	0.197	1.035 (0.416–2.578)	0.941
CCI	1.121 (1.071–1.173)	< 0.001	1.125 (1.054–1.2)	< 0.001	1.112 (1.047–1.181)	< 0.001	1.057 (0.979–1.142)	0.156	1.056 (0.936–1.191)	0.374	1.052 (0.953–1.162)	0.316
Year of endometrial cancer diagnosis	1.112 (1.064–1.162)	< 0.001	1.116 (1.048–1.189)	0.001	1.101 (1.039–1.167)	0.001	1.124 (1.057–1.194)	< 0.001	1.141 (1.041–1.25)	0.005	1.11 (1.026–1.201)	0.009
Types of primary treatment ^b,c^												
Surgery ^d^	1.081 (0.821–1.424)	0.579	1.166 (0.784–1.732)	0.448	1.033 (0.718–1.486)	0.862	1.111 (0.739–1.671)	0.614	1.202 (0.645–2.239)	0.563	1.122 (0.657–1.917)	0.672
Radiotherapy	0.849 (0.368–1.957)	0.701	1.196 (0.423–3.382)	0.736	0.72 (0.223–2.33)	0.584	1.383 (0.487–3.927)	0.542	2.516 (0.714–8.866)	0.151	1.174 (0.272–5.076)	0.83
Chemotherapy	2.334 (1.38–3.949)	0.002	1.944 (0.852–4.437)	0.114	2.742 (1.424–5.278)	0.003	2.532 (1.291–4.966)	0.007	1.452 (0.411–5.137)	0.563	3.366 (1.496–7.576)	0.003
Hormone therapy	2.073 (1.356–3.17)	0.001	1.837 (0.973–3.469)	0.061	2.086 (1.19–3.657)	0.01	0 (0-Infinite)	0.991	0 (0-Infinite)	0.994	0 (0-Infinite)	0.994

*CCI* Charlson comorbidity index; *CI* confidence interval; *DVT* deep vein thrombosis; *HIRA* health insurance review & assessment service; *HR* hazard ratio; *SES* socioeconomic status; *PE* pulmonary embolism; *VTE* venous thromboembolism

^a^ The reference was mid- or high- SES

^b^ Primary treatments refers to the first cancer treatments

^c)^ The reference was no treatment. In patients that received no treatment, the incidence of VTE after diagnosis of endometrial cancer was evaluated

^d^ Neoadjuvant chemotherapy followed by laparotomy or laparoscopy was considered as surgery

^e^ The data were adjusted for all risk factors (age per 5 years, SES, CCI, year of endometrial cancer diagnosis, and types of primary treatment)

During the first 6 months, the risk of VTE significantly increased according to increased age and year of endometrial cancer diagnosis (age: HR, 1.147; 95% CI, 1.066–1.235; *P* < 0.001) (year of endometrial cancer diagnosis: HR, 1.124; 95% CI, 1.057–1.194; *P* < 0.001). Low SES, and CCI were not risk factors of VTE. Chemotherapy (as the only treatment) was associated with a significant increase in VTE risk, especially for PE, as compared with no treatment after adjustment for confounders (VTE: HR, 2.532; 95% CI, 1.291–4.966; *P* = 0.007) (PE: HR, 3.366; 95% CI, 1.496–7.576; *P* = 0.003) (Table 3).

### Incidence of VTE according to use of the prophylactic anticoagulants after primary treatments

During the total follow-up period, 14.5% of women with endometrial cancer received prophylactic anticoagulants. Moreover, 6.2% of women with VTE did not undergo prophylactic anticoagulant therapy (4.5% of DVT and 7% of PE cases) and 93.8% underwent prophylactic anticoagulant therapy (95.5% of DVT and 93% of PE cases). VTE occurred in 0.1% (0% of DVT and 0.1% of PE cases) of women that did not receive prophylactic anticoagulant therapy and in 8.8% (4.5% of DVT and 4.9% of PE cases) of women that underwent prophylactic anticoagulant therapy (Additional file 3).

Prescribed prophylactic anticoagulants, in decreasing order of frequency, were UFH (78.0%), DOAC (16.5%), warfarin (15.7%), LMWH (11.9%), and fondaparinux (0.1%). The most frequently used therapeutic anticoagulant was DOAC (59.1%), and an inferior vena cava filter was used in 5.9% of women with VTE (Additional file 4).

## Discussion

Our study demonstrated the highest incidence of VTE during the total follow-up period and during the first 6 months occurred in women with endometrial cancer administered chemotherapy as a primary cancer treatment. The monthly incidence of VTE decreased with time during the first year in the no treatment, surgery, and hormone therapy groups but did not change in the radiotherapy or chemotherapy groups. Furthermore, the risk of VTE was greater for women that underwent chemotherapy or hormone therapy than for those that received no treatment.

Reported incidences of VTE in women with endometrial cancer range between 0.35 and 8% [6, 7, 9–13, 15, 16, 25]. In the present study, VTE incidences agreed with those previously reported (1.4% during the total follow-up period and 0.5% during the first 6 months). In one cohort study, the absolute rate of VTE in women with endometrial cancer (*n* = 1958) decreased over time after cancer diagnosis [26]. In another two cohort studies, the incidence of VTE decreased over time for 30 (*n* = 175) and 90 days (*n* = 4158) following gynecologic cancer surgery [27, 28], and in a fourth cancer cohort study conducted on a claims database (*n* = 17,284), the incidence of VTE decreased over time during the 12 months following the initiation of chemotherapy [18]. Similarly, we found that in the no treatment, surgery, and hormone therapy groups, the incidence of VTE decreased with time during the first year after primary cancer treatment initiation. However, in our radiotherapy and chemotherapy groups, no longitudinal change in the incidence of VTE was observed. Further studies are needed to clarify VTE occurrence trends with respect to time for different primary cancer treatment types.

Previous studies have reported incidences of 0.35–8% for VTE in women with endometrial cancer that have undergone surgery (open or minimally invasive surgery including staging surgery) [6, 7, 9–13, 15, 16], and incidences of 0–13.8% in women with ovarian, cervical, or endometrial cancer that have undergone surgery [5–16]. In one cohort study, VTE occurred in 1.2% of women that had undergone gynecologic brachytherapy for cervical, endometrial, or vaginal cancer (*n* = 329) [17], and in a claims database analysis of women with ovarian cancer (*n* = 1880), the incidence of VTE was reported to be 11% during the first year after the initiation of chemotherapy [18]. In a cohort study performed on women with cervical cancer (*n* = 798), the incidence of VTE was 33.8% among women that received chemotherapy, and chemotherapy was found to be a risk factor of VTE [19]. In another cohort study performed on women with ovarian cancer (*n* = 328), non-receipt of treatment for cancer was a risk factor of VTE [29]. In our study, the incidence of VTE was highest for chemotherapy (2.4%) followed in decreasing order of frequency by hormone therapy (1.6%), no treatment (1.3%), surgery (1.3%), and radiotherapy (1.1%) during the total follow-up period, and chemotherapy (1.6%), radiotherapy (0.7%), surgery (0.6%), no treatment (0.6%), and hormone therapy (0%) during the first 6 months of follow-up. Furthermore, compared with women that received no treatment, VTE risk, especially for PE, was significantly higher in women that received chemotherapy or hormone therapy during the total follow-up period and was significantly higher for women that underwent chemotherapy at six-month follow-up visits. It has been reported that sudden fatal PE event is a leading cause of VTE-related deaths in medical and surgical patients [30]. Current guidelines recommend that anticoagulant treatment for VTE prophylaxis during systemic chemotherapy may be considered for ambulatory cancer patients at intermediate or high risk of VTE (Khorana score ≥ 2) [4, 31, 32]. Furthermore, gynecologic cancers are considered high risk by the Khorana predictive model for chemotherapy-associated VTE [32], and women with endometrial cancer that receive chemotherapy as a primary treatment usually have advanced disease and are at high risk for VTE [4, 20]. Therefore, our findings that chemotherapy as a primary therapeutic modality is a risk factor of VTE and associated with the highest incidence of VTE support the use of anticoagulants to prevent chemotherapy-associated VTE in women with endometrial cancer that receive chemotherapy as a primary treatment.

In a study of women with endometrial cancer treated by surgical staging (*n* = 714), the incidence of VTE was greater in women older than 60 years than in women younger than 60 years (10.7% vs 7.1%) [11]. Similarly, in another endometrial cancer cohort (*n* = 1958), the absolute rate of VTE was higher in women over 60 years than in women under 60 years (13 per 1000 person years vs 7 per 1000 person years) [26]. In our study, regardless of follow-up duration after primary treatments initiations, women with VTE were older than women without, and the risk of VTE increased with age. In addition, we observed changes in VTE occurrence according to primary treatment type and age. Interestingly, for all types of primary treatment, except hormone therapy, we found the incidence of VTE decreased from 70 or 75 years of age. We suggest this finding be confirmed by further study.

In our study, 14.5% of women with endometrial cancer underwent prophylactic anticoagulant treatment during the total follow-up period, and the incidence of VTE were higher in those that received prophylactic treatment. We believe that women exposed to VTE risk factors such as old age, high CCI, radical hysterectomy, chemotherapy, or hormone therapy are likely to receive prophylactic anticoagulant treatments more frequently.

This nationwide, population-based cohort study provides more data than multicenter studies. Furthermore, it is the first to evaluate the incidence and risk of VTE in a large cohort according to primary treatment in women with a diagnosis of endometrial cancer and to examine relationships between chemotherapy or hormone therapy and VTE. Limitations of this study are associated with analysis using claims data. First, diseases were defined using diagnostic and prescription codes, and medical records were not reviewed, and thus, a few women coded incorrectly may have been misdiagnosed. Second, women that received prescriptions for anticoagulants less than twice were not considered to have VTE. Based on the coverage provided by the Korean national health insurance system, women with VTE are usually treated regardless of symptoms, and thus, we believe only a small proportion of those affected were not treated for VTE. Third, women with VTE before endometrial cancer diagnosis or initiation of primary cancer treatments were not included. If diagnosis of VTE or endometrial cancer and initiation of primary cancer treatment occurred within a short time frame, it is possible that the order of diagnosis was inaccurate. Finally, we did not evaluate relationships between VTE and BMI or stage or histologic type of endometrial cancer because the HIRA dataset did not provide this information. Nonetheless, our study indicates primary treatment presents a greater risk of VTE than no treatment, which might be because of the dependence of treatment modality on stage.

This retrospective analysis of claims data presents the incidence and risk of VTE and time to VTE occurrence according to primary treatment type in women with endometrial cancer. We hope that the results of this study help guide the prophylaxis and treatment of VTE, which is a serious complication in women with endometrial cancer.

## Supplementary Information

**Additional file 1: Supplemental Figure 1**. Incidences of VTE according to age in women with endometrial cancer (based on HIRA claims data for 2009–2018). In women that received no treatment, the incidence of VTE after diagnosis of endometrial cancer was evaluated.

**Additional file 2: Supplemental Table 1**. Incidences of VTE according to age in women with endometrial cancer (based on HIRA claims data for 2009–2018). In women that received no treatment, the incidence of VTE after the diagnosis of endometrial cancer was evaluated.

**Additional file 3: Supplemental Table 2**. Incidences of VTE according to prophylactic anticoagulant use in women with endometrial cancer (based on HIRA claims data for 2009–2018).

**Additional file 4: Supplemental Table 3**. Methods of VTE prophylaxis and treatment in women with endometrial cancer (based on HIRA claims data for 2009–2018).

## Data Availability

The data that support the findings of this study are available from the Health Insurance Review and Assessment Service (HIRA) but restrictions apply to the availability of these data, which were used under license for the current study, and so are not publicly available. Data are however available from the authors upon reasonable request and with permission of the HIRA.

## Abbreviations

VTE: Venous thromboembolism
DVT: Deep vein thrombosis
PE: Pulmonary embolism
TH: Total hysterectomy
RH: Radical hysterectomy
BSO: Bilateral salpingo-oophorectomy
EBRT: External beam radiation therapy
HIRA: Health insurance review and assessment service
ICD-10: The 10th revision of the international statistical classification of diseases and related health problems
SES: Socioeconomic status
CCIs: Charlson comorbidity indices
CCRT: Concurrent chemoradiotherapy
DOAC: Direct oral anticoagulants
LMWH: Low molecular weight heparin
UFH: Unfractionated heparin
