# Supplementary material for: Incidence and risk of venous thromboembolism according to primary treatment type in women with endometrial cancer: a population-based study
Source: BMC Cancer. 2021 Oct 30;21:1166. doi: 10.1186/s12885-021-08853-x (PMC8557555; doi:10.1186/s12885-021-08853-x)
Supplement: Supplementary file 2 — Additional file 2: Supplemental Table 1. Incidences of VTE according to age in women with endometrial cancer (based on HIRA claims data for 2009–2018). In women that received no treatment, the incidence of VTE after the diagnosis of endometrial cancer was evaluated. [file 12885_2021_8853_MOESM2_ESM.docx]

|  |  |  | The total follow-up period | | | | | | | | | | | | | | Total cases |
| --- | --- | --- | --- | --- | --- | --- | --- | --- | --- | --- | --- | --- | --- | --- | --- | --- | --- |
|  |  | Age (years) | ~15 | ~20 | ~25 | ~30 | ~35 | ~40 | ~45 | ~50 | ~55 | ~60 | ~65 | ~70 | ~75 | >75 | (n=26,256) |
| Primary treatments ^a)^ |  |  |  |  |  |  |  |  |  |  |  |  |  |  |  |  |  |
| No treatment ^b)^ | VTE cases |  | 0 | 0 | 0 | 0 | 1 | 0 | 5 | 7 | 14 | 10 | 9 | 5 | 10 | 9 |  |
|  | Number of women |  | 1 | 7 | 29 | 74 | 168 | 288 | 516 | 854 | 977 | 709 | 424 | 344 | 330 | 617 | 5,338 |
|  | VTE cases per 10,000 women |  | 0 | 0 | 0 | 0 | 60 | 0 | 97 | 82 | 143 | 141 | 212 | 145 | 303 | 146 |  |
| Surgery ^c)^ | VTE cases |  | 0 | 0 | 1 | 2 | 1 | 3 | 8 | 23 | 36 | 43 | 40 | 42 | 18 | 14 |  |
|  | Number of women |  | 0 | 0 | 21 | 89 | 308 | 682 | 1487 | 2456 | 3737 | 3546 | 2266 | 1447 | 871 | 707 | 17,617 |
|  | VTE cases per 10,000 women |  | 0 | 0 | 476 | 225 | 32 | 44 | 54 | 94 | 96 | 121 | 177 | 290 | 207 | 198 |  |
| Radiotherapy | VTE cases |  | 0 | 0 | 0 | 0 | 0 | 0 | 0 | 0 | 2 | 1 | 0 | 1 | 1 | 1 |  |
|  | Number of women |  | 0 | 0 | 0 | 2 | 8 | 20 | 33 | 66 | 113 | 119 | 59 | 40 | 42 | 48 | 550 |
|  | VTE cases per 10,000 women |  | 0 | 0 | 0 | 0 | 0 | 0 | 0 | 0 | 177 | 84 | 0 | 250 | 238 | 208 |  |
| Chemotherapy | VTE cases |  | 0 | 0 | 0 | 0 | 1 | 1 | 2 | 1 | 2 | 3 | 3 | 3 | 1 | 1 |  |
|  | Number of women |  | 1 | 1 | 4 | 8 | 11 | 36 | 69 | 123 | 123 | 151 | 98 | 62 | 24 | 30 | 741 |
|  | VTE cases per 10,000 women |  | 0 | 0 | 0 | 0 | 909 | 278 | 290 | 81 | 163 | 199 | 306 | 484 | 417 | 333 |  |
| Hormone therapy | VTE cases |  | 0 | 0 | 1 | 0 | 1 | 0 | 2 | 6 | 2 | 3 | 4 | 4 | 3 | 6 |  |
|  | Number of women |  | 2 | 11 | 81 | 218 | 400 | 286 | 160 | 149 | 169 | 140 | 116 | 80 | 79 | 119 | 2,010 |
|  | VTE cases per 10,000 women |  | 0 | 0 | 123 | 0 | 25 | 0 | 125 | 403 | 118 | 214 | 345 | 500 | 380 | 504 |  |

**Additional file 2: Supplemental Table 1. Incidences of VTE according to age in women with endometrial cancer (based on HIRA claims data for 2009-2018).**

HIRA, Health Insurance Review & Assessment Service; VTE, venous thromboembolism.

^a)^ Primary treatments refers to the first cancer treatments.

^b)^ Incidence of VTE after diagnosis of endometrial cancer was evaluated.

^c)^ Neoadjuvant chemotherapy followed by laparotomy or laparoscopy was considered as surgery.
