# Supplementary material for: Incidence and risk of venous thromboembolism according to primary treatment type in women with endometrial cancer: a population-based study
Source: BMC Cancer. 2021 Oct 30;21:1166. doi: 10.1186/s12885-021-08853-x (PMC8557555; doi:10.1186/s12885-021-08853-x)
Supplement: Supplementary file 3 — Additional file 3: Supplemental Table 2. Incidences of VTE according to prophylactic anticoagulant use in women with endometrial cancer (based on HIRA claims data for 2009–2018). [file 12885_2021_8853_MOESM3_ESM.docx]

**Additional file 3: Supplemental Table 2. Incidences of VTE according to prophylactic anticoagulant use in women with endometrial cancer (based on HIRA claims data for 2009-2018).**

|  | Total | VTE | | | DVT | | | PE | | |
| --- | --- | --- | --- | --- | --- | --- | --- | --- | --- | --- |
|  | (n=26,256) | No | Yes | *P* value | No | Yes | *P* value | No | Yes | *P* value |
|  |  | (n=25,899) | (n=357) |  | (n=26,078) | (n=164) |  | (n=26,055) | (n=201) |  |
| Pharmacologic thromboprophylaxis |  |  |  | <0.001 |  |  | 0.001 |  |  | <0.001 |
| No | 22,445 (85.5) | 22,423 (99.9) | 22 (0.1) |  | 22,437 (100) | 8 (0) |  | 22,431 (99.9) | 14 (0.1) |  |
| Yes | 3,811 (14.5) | 3,476 (91.2) | 335 (8.8) |  | 3,641 (95.5) | 170 (4.5) |  | 3,624 (95.1) | 187 (4.9) |  |

DVT, deep vein thrombosis; PE, pulmonary embolism; VTE, venous thromboembolism.

All values ​​are expressed as number (%).
