# Supplementary material for: Incidence and risk of venous thromboembolism according to primary treatment type in women with endometrial cancer: a population-based study
Source: BMC Cancer. 2021 Oct 30;21:1166. doi: 10.1186/s12885-021-08853-x (PMC8557555; doi:10.1186/s12885-021-08853-x)
Supplement: Supplementary file 4 — Additional file 4: Supplemental Table 3. Methods of VTE prophylaxis and treatment in women with endometrial cancer (based on HIRA claims data for 2009–2018). [file 12885_2021_8853_MOESM4_ESM.docx]

**Additional file 4: Supplemental Table 3. Methods of VTE prophylaxis and treatment in women with endometrial cancer (based on HIRA claims data for 2009-2018).**

|  | Prophylaxis (n=3,811) | Treatment (n=357) |
| --- | --- | --- |
| UFH | 2,972 (78.0) | 172 (48.2) |
| LMWH | 452 (11.9) | 74 (20.7) |
| Fondaparinux | 3 (0.1) | 0 (0) |
| Warfarin | 598 (15.7) | 141 (39.5) |
| DOAC | 629 (16.5) | 211 (59.1) |
| Aspirin |  | 159 (44.5) |
| Thrombectomy |  | 3 (0.8) |
| Thromboplasty |  | 0 (0) |
| Thrombolysis |  | 2 (0.6) |
| IVC filter |  | 21 (5.9) |

DOAC, direct oral anticoagulants; IVC, Inferior Vena Cava; LMWH, low molecular weight heparin; UFH, unfractionated heparin; VTE, venous thromboembolism.

All values ​​are expressed as number (%).
